# Biofabrication of Nanosilver From *Punica granatum* Peel Extract and Their Anticoagulant Applications

**DOI:** 10.1155/2024/6623228

**Published:** 2024-09-26

**Authors:** Randa Mohammed Dhahi

**Affiliations:** Department of Biology College of Education Al-Iraqia University, Baghdad, Iraq

**Keywords:** agrowaste management, biocompatibility, green biosynthesis, pomegranate peel extract, silver nanoparticle

## Abstract

For utilizing biodegradable waste as a natural source for nanofabrication, this study was designed to highlight a simple, sustainable, safe, environmentally friendly, and energy consumption reduction waste management approach using hot aqueous extract of *Punica granatum* (pomegranate) peel waste (PPE) to biosynthesize silver nanoparticles (PPE-AgNPs). The fabrication of biosynthesized nanosilver was confirmed by UV–visible spectroscopy, scanning electron microscopy (SEM), energy-dispersive X-ray spectroscopy (EDX), X-ray diffraction (XRD), and atomic force microscope (AFM). The initial pale brown color change upon adding silver nitrate to PPE confirmed bioreduction. For PPE, the absorption spectrum for UV–vis spectroscopy in the visible light region was 230–290 nm, while for PPE-AgNPs, the graph shows that surface plasmon resonance (SPR) spectrum for nanosilver at 360–460 nm. The XRD analysis proved that the PPE-AgNPs were crystalline in nature. The SEM micrograph revealed that silver nanoparticles were sphere-shaped, homogenous accumulations with particle size in the range of 21.63–30.97 ± 0.4 nm. The EDX data analysis also proved the presence of a sharp peak of silver element with 8.83% weight at 3 keV. The 3D AFM images of Ag nanoparticles illustrated that the diameter is around 7.20–14.80 nm with a median of 7.16 ± 1.3 nm and the root mean square (RMS) value corresponds to 1.40 ± 0.4 nm. The PPE-AgNPs efficiently exhibited a potent antioxidant and dose-dependent DPPH inhibition action. Visual and microscopic observations of fresh human blood when treated with 25, 50, 75, and 100 *μ*g/mL of PPE-AgNPs were proven to be biocompatible with no morphological changes and no coagulation. This study predicts that PPE can be utilized to synthesize biocompatible nanosilver.

## 1. Introduction

Food waste is a humanitarian and environmental issue. Additionally, vegetable and fruit waste can be a natural source rich in biomolecules and phytochemicals used in uncountable uses in pharmaceutical and medical therapy [1]. These biomolecules could serve as capping and bioreduction agents for the production of metallic-based nanoparticles with distinctive physicochemical properties [2]. Inadequate reports are available to describe agrowaste in ecofriendly biological methods for nanoparticle synthesis. Several studies have stated that nanoparticle production uses huge sources of plants yearly, but very few studies have shown how specifically fruit waste can be valorized into important products used by humans, such as nanoparticles [3]. The use of biodegradable agrowaste to biosynthesize nanomaterials could have significant environmental benefits. Nanotechnology is an exciting area that could possibly tackle the problem of nonbiodegradable waste management as well as nanomaterial synthesis with biomedical applications. Recently, nanotechnology has gained a remarkable impact in industrial and medical endeavors. Their utilization is widespread within the food and drug industries [4]. Nanotechnology concentrates on nanoscale particle fabrication with unique applications in the chemistry, physics, biology, and medicine fields [5]. Silver nanoparticles exhibit desirable physicochemical properties such as size, shape, surface-to-volume ratio, crystallinity, surface plasmon resonance (SPR), solubility, and biocompatibility that make them applicable in biomedicine [6, 7]. Lately, the limitations of traditional therapy and the challenges demonstrated by silver nanoparticle–based technologies proved the silver nanoparticles' importance for biological applications [8, 9]. Nevertheless, nanomaterials are produced via two main approaches: top-down or bottom-up; the bottom-up method is tunable, high yield, and cheaper to follow as compared with the top-down approach [10]. Because of plant availability, green synthesis of nanoparticles was preferred in contrast to other biological systems [11, 12]. Plant extracts have served as an important biomolecule reservoir for nanoparticle production [13]. Pomegranate *Punica granatum* and its active constituents were known as one of the most popular, with valuable fruits as well as their pharmacological characteristics as antioxidants [14], antibacterial [15], anticancer, and antiaging actions [16]. According to our knowledge, the action of AgNPs fabricated from *P. granatum* peel waste (PPE) extract on blood hemolysis and coagulation has still been little researched. So, this study was for utilization of agrowaste resources of Punica granatum peel (PPE) waste as value-added natural agent for production of green silver nanoparticles as well as study the biocompatibility and anticoagulation action.

## 2. Materials and Method

### 2.1. Pomegranate Aqueous Peel Extraction and Phytochemical Analysis

The ripened pomegranate fruit was purchased from a marketplace in Baghdad, Iraq. For cleaning, pomegranate fruit was rinsed for 30 min with filtered water, then peels were manually detached. The peels were dried at 30°C on filter paper for 4 days. Then, it was chopped and kept at room temperature (27°C). Later, 100 g of dried peel pieces were mixed with 1 L of deionized water and then boiled in a water bath for 15 min. Whatman No. 1 filter paper was used for the filtration of the resulting solution to get a pure and clear extract [17]. The filtrates thus obtained were stored at 4°C for gas chromatography-mass (GC-mass) phytochemical analysis and nanosilver biofabrication and characterization.

GC-MS analysis was carried out with the Shimadzu QP-2010T instrument, which constituted an autosampler and gas chromatography interfaced to a mass spectrometer using the following conditions: capillary column −624 ms (30 m × 0.32 mm × 1.8 m) operating in an electronic mode at 70 eV; helium (99.99%) was used as the carrier gas at a constant flow of 1.491 mL/min and injection volume of 1.0 mL, injector temperature of 140°C, and ion source temperature of 200°C. The oven temperature was set at 45°C. Mass spectra were taken at 70 eV [18].

### 2.2. Nanosilver Biofabrication

About 5 mL of PPE was added to 50 mL of 1 mM aqueous solution of silver nitrate (AgNO_3_) in the reaction vessel and kept at room temperature; a magnetic stirrer at 27°C was used for constant mixing of PPE for 35 min to minimize the silver ion formation. Then, the resulting solution was stored in a dark room all night at 27°C to ensure bioreduction. The PPE-AgNPs could be produced by centrifuging at 10,000 rpm for 15 min; later, the supernatant was filtered and stored at 4°C for nanosilver bioproduction confirmation and characterization [19].

### 2.3. Characterization of PPE-AgNPs

Physical and chemical characterization techniques were followed to study and confirm the biosynthesis of nanosilver. The nanoparticle formation was analyzed using a UV–visible spectrophotometer, and the reduction of Ag ions to silver nanoparticles was monitored by measuring the UV–visible spectra of samples after diluting the sample with deionized water many times. The spectra of the silver nanoparticle solution were observed using a UV–vis spectrophotometer equipped with matched quartz cells (Shimadzu model no. UV1601) from 200 to 800 nm. Deionized water was used as a blank to correct the baseline [20].

To examine the crystalline nature of PPE-AgNPs, the XRD pattern was followed. Scanning electron microscopy (SEM) equipped with energy dispersive X-ray spectroscopy (EDX) was used to study the size and morphology and to reveal the presence of the elements in the biofabricated AgNPs and the morphology of nanosilver. The sample was prepared by dropping a small amount of the sample on a carbon-coated copper grid and then observed by SEM (Hitachi, S-3000). The extra solution was removed using a blotting paper; then, the film placed on the SEM grid was allowed to dry by putting it under a lamp for 5 min. A three-dimensional (3D) image by atomic force microscope (AFM) uncovered the size distribution and topology of PPE-AgNPs [21].

### 2.4. In Vitro Antioxidant Activity of PPE-AgNPs

DPPH or 2,2-diphenyl-1-picrylhydrazyl is a free radical used to study the PPE-AgNPs scavenging action at different concentrations (25, 50, 75, and 100 *μ*g/mL). Initially, 1 mL of 1 mM DPPH was dissolved in 50 mL of methanol, mixed by vortex, and incubated for 35 min, then all vessels were kept in darkness. The absorbance was studied using a multireader at 517 nm. The negative and positive controls were DPPH and ascorbic acid, respectively. Methanol was used as a standard for this assay. The percent inhibition activity was calculated according to Equation (1).

(1) $$\begin{array}{c} \text{Scavenging }\left(\%\right)=\frac{Ac-As}{Ac}\times 100 \end{array}$$

where *A*_*C*_ represents the absorbance of the control and *A*_*s*_ is the absorbance of DPPH with sample [22].

### 2.5. Antihemolysis and Biocompatibility Study of PPE-AgNPs

Freshly collected blood samples of healthy volunteers without any anticoagulant agents were used for the nanosilver anticoagulation study. About 2.5 mL of 100 mM of phosphate-buffered saline (PBS with pH = 7.0) was mixed with 200 *μ*L of blood sample. Centrifuging at 1000 rpm for 10 min was followed for red blood cell (RBC) separation, then washed with PBS many times, and the resultant pellet of RBC was dissolved in 10 *μ*L of PBS and used for further study [23]. The following concentrations (25, 50, 75, and 100 *μ*g/mL) of PPE-AgNPs were used to investigate biocompatibility, then incubated with blood samples at 35°C for 30 min. The stability of PPE-AgNPs was evaluated by adding 100 *μ*g/mL of PPE-AgNPs to normal saline (NS) and distilled water (DW) as positive and negative controls, respectively [24]. Then, all samples were centrifuged at 3000 rpm for 15 min, and the supernatant was collected to measure at 545 nm absorbance. Likewise, blood samples were tested with PPE-AgNPs at 25, 50, 75, and 100 *μ*g/mL after 4 h and examined under a light compound microscope at 40x magnification for any morphologic changes.

### 2.6. Anticoagulant and Thrombolytic Property Action of PPE-AgNPs

The anticoagulant property of the biofabricated nanosilver was assessed according to [25] with a slight modification. About 500 *μ*L (25 *μ*g/mL) of PPE-AgNPs was spread on a clean glass slide, then added 1 mL of freshly collected human blood. The resultant mix was incubated at 25°C for 10 min to allow clot formation and to detect any coagulation. Another blood sample was spread and treated with DW as a negative control.

## 3. Results

**Figure 1:**
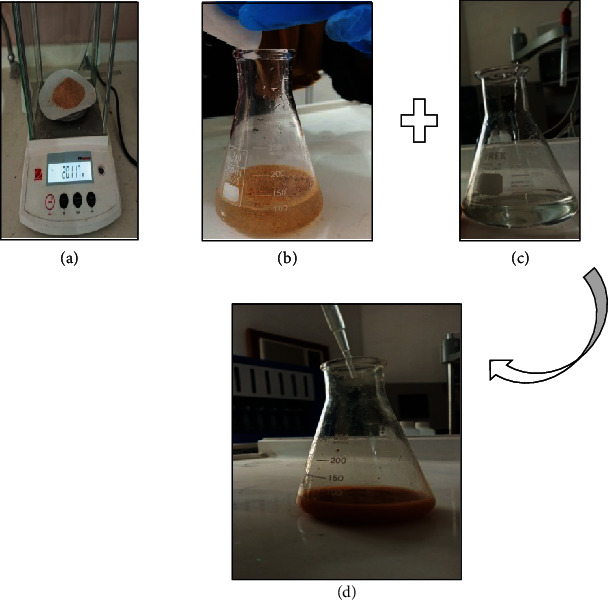
Preliminary indication for biosynthesis of nanosilver. (a) Pomegranate peel powder, (b) pomegranate peel aqueous extraction, (c) aqueous silver nitrate solution, and (d) bioreduction of PPE based on color change from dark brown to pale brown.

The GC-mass for an aqueous extract of pomegranate peel of Iraqi origin (Figure 1(b)) has uncovered the following phytochemicals: gallic acid as a polyphenol with a ratio of 42.8%, followed by ellagic acid (14.7%), the third constituent is quercetin (9.7%) as a flavonoids, then punicalagin (5.18%), followed by the geraniol with a ratio of 4.7%, and furfural (3.3%). The other major phytochemical constituent are .gamma.-Sitosterol (3.2%), Ascorbic acid (2.0 %), caffeic acid (1.9%); that was 87.5 %, the ratio of other components are small compared with the major phytochemical constituent, varying carbohydrate anthocyanidins, triterpenoids, steroids, glycosides, and esters in very trace amounts. Many studies have shown that the PPE exhibited almost the same phytochemical [26–30]. The pomegranate peel waste aqueous extract used as a capping and bioreduction agent for nanosilver particle biosynthesis was confirmed firstly by the formation of whitish brown production when mixing with AgNO_3_ dropwisely (Figures 1(c) and 1(d)). The colorless silver nitrate solution turned to a whitish brown color (Figure 1(c)) as a clue for the bioreduction of nanosilver based on the color change due to SPR excitation.

Pomegranate peel is a natural and abundant source of phytochemical compounds like polyphenols such as gallic acid, flavonoids, and tannins [31]. The carboxylic and free hydroxyl group phytochemicals found in the pomegranate peels might combine with the surface of silver ions and stimulate nanosilver production [32].

**Figure 2:**
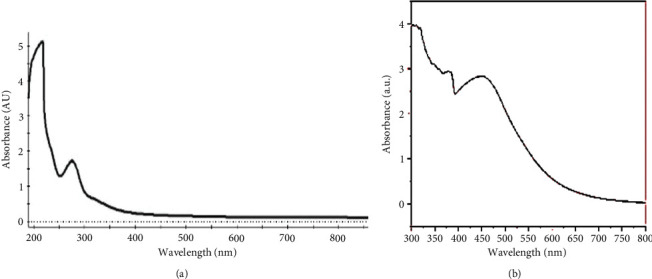
UV–vis spectrophotometer for (a) PPE and (b) biosynthesized silver nanoparticle PPE-AgNPs.

The biosynthesis of silver nanoparticles was confirmed by physical and chemical characterization. The synthesis and stability of silver-based nanoparticles were examined with UV–vis spectroscopy at wavelengths of 200–800 nm (Figures 2(a) and 2(b)). For the PPE, the absorption spectrum was 230–290 nm (Figure 2(a)), while for PPE-AgNPs, it was 360–460 nm (Figure 2(b)). In general, the typical SPR band for silver nanoparticles was in the range of 400–500 nm [33]. The SPR controlled the optical absorption spectra of metallic-based nanoparticles.

**Figure 3:**
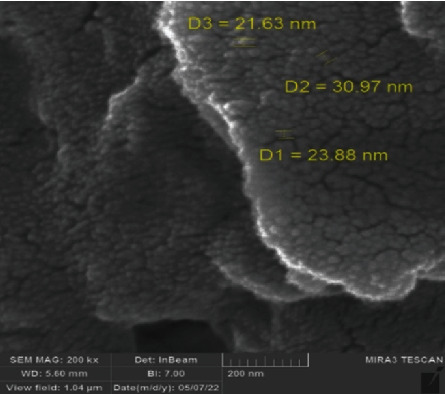
SEM micrograph for PPE-AgNPs from pomegranate peels.

SEM results distinctly depict the morphology and size of the biosynthesized nanosilver. The size was in the range of 21.63–30.97 ± 0.4 nm with sphere-shaped, homogenous accumulation particles (Figure 3).

**Figure 4:**
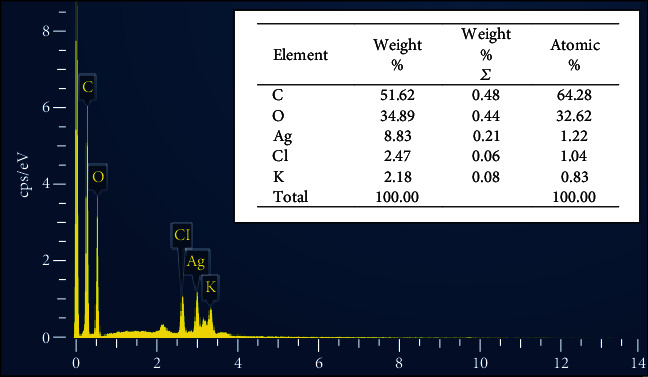
EDX analysis of synthesized nanosilver from *P. granatum* peel extract.

This result was comparable to an earlier report that emphasized spherical-shaped nanosilver with an average particle size of 59 nm produced using PPE [34]. Methanolic and water extracts of pomegranate peel have been investigated for the silver nanoparticle's biosynthesis with a small NP size (20 nm) for cancer treatment [35]. Another report demonstrated that the hard pericarp of pomegranate extract was used for zinc oxide (ZnO) nanoparticle synthesis as an antibacterial agent with sizes 18 and 30 nm [36]. Lately, a report synthesized bimetallic zinc and silver nanoparticles from pomegranate extract with a size of 15 nm for antibacterial therapeutic benefits [37]. Data from the EDX analysis investigate additional information about the elements found in the biosynthesized nanosilver, as illustrated in Figure 4. The element weight percent identified in the results included silver (Ag) (8.83%) at 3 keV as well as oxygen (O) (34.89%), carbon (C) (51.62%), chlorine (Cl) (2.47%), and potassium (K) (2.18%). These results suggested that the surface of nanosilver was enclosed by biomolecules, each of them matched to a small percentage of the total mass of the pomegranate plant extract (act as a capping ligand). The obtained EDX results are close to the findings of [38]. Chlorine signal in EDX analysis could be from the mount step during the sample preparation (using epoxy).

**Figure 5:**
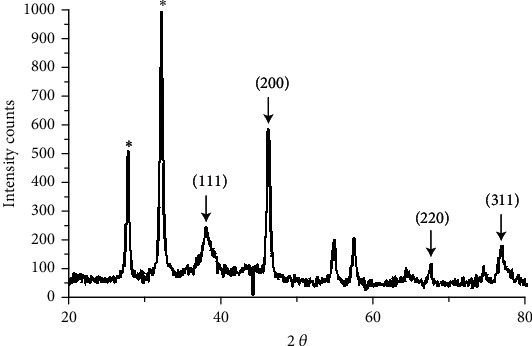
XRD pattern of biofabricated nanosilver from *P. granatum* peel extract.

Figure 5 illustrates the XRD pattern of biofabricated nanosilver. The Braggs reflections observed at 2*θ* = 38.68°, 46.46°, 65.55°, and 78.31° correspond to (111), (200), (220), and (311) lattice planes, correspondingly, which can be approved of the cubic crystal nature of nanosilver. This conclusion was approved by XRD patterns presented in the Joint Committee on Powder Diffraction Standards (JCPDS) database (No. 04-0783). The unidentified crystalline peaks (^∗^) detected are equivalent to Ag oxides.

**Figure 6:**
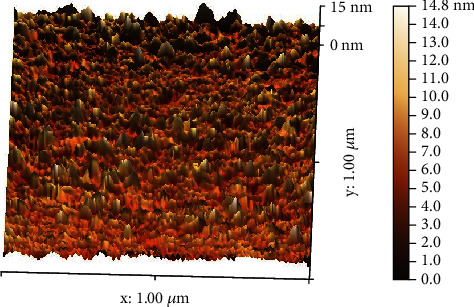
3D AFM image of biosynthesized PPWE-AgNPs.

The 3D AFM images of nanosilver are illustrated in Figure 6, and the diameter was around 7.20–14.80 nm with a median of 7.16 ± 1.3 nm, and the root mean square (RMS) value corresponds to 1.40 ± 0.4 nm. The analysis proved that the synthesized PPE-AgNPs were less than 50 nm, correlating with the results of SEM analysis. AFM analyses are appropriate for the size characterization of nanoformulation materials in pharmaceutical research [39].

**Figure 7:**
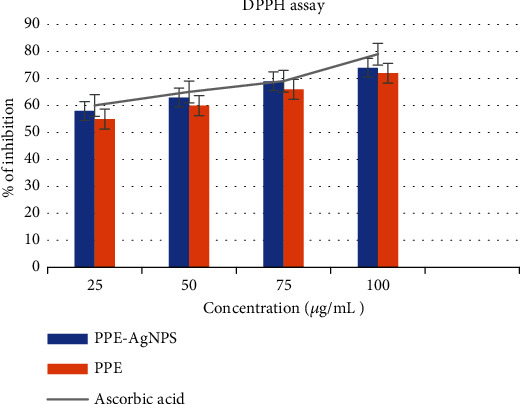
DPPH free radical scavenging action of the PPE and biofabricated PPWE-AgNPs.

Antioxidant properties of PPE and biosynthesized PPE-AgNPs were evaluated against free radicals DPPH as presented in Figure 7. The PPE-AgNPs efficiently exhibited a dose-dependent DPPH inhibition activity.

The inhibition percentages were 58%, 63%, 69%, and 74% of the biofabricated PPE-AgNPs that were raised with concentrations 25, 50, 75, and 100 *μ*g/mL, respectively. However, the inhibition percentage of PPE was 55%, 60%, 66%, and 72% for concentrations 25, 50, 75, and 100 *μ*g/mL, respectively.

The in vitro DPPH assay approved notable antioxidant action of biofabricated PPE-AgNPs which reveals the important role of phytochemicals in PPE, specifically phenolic compounds in eradicating free radicals. It was confirmed that the inhibition action increased as the concentrations of biofabricated AgNPs, PPE, and ascorbic acid increased. The phenolic content of the PPE that is capped on nanosilver could boost the antioxidant action [40]. Free radicals have an adverse effect on human health because they contain a complex antioxidant resistance system of nonenzymatic and enzymatic [41]. Many diseases like diabetes mellitus, Parkinson's disease, neural disorder, and age-related diseases were attributed to free radicals [42]. A previous study by [43] showed that the phenolic contents and flavonoids were higher in peel extract than in pulp extract.

The phenolics included in pomegranate peel extract may be the source of its strong antioxidant action. PPE-AgNPs from pomegranate fleshy pericarp had shown antioxidant activity [44].

**Figure 8:**
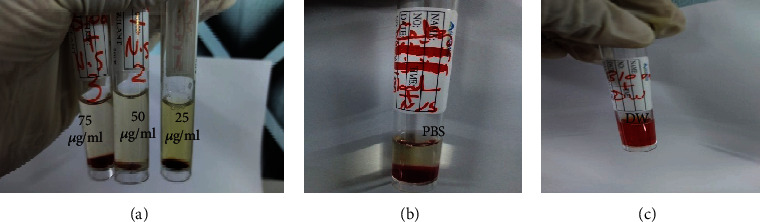
Visual observation for the in vitro PPE-AgNP hemolytic activity. (a) RBC treated with different concentrations (25, 50, and 75 *μ*g/mL) of PPE-AgNPs, (b) RBC treated with phosphate-buffered saline (PBS) as a positive control, and (c) RBC treated with distilled water as a negative control.

Figure 8 represents PPE-AgNP solution in concentrations 25, 50, and 75 *μ*g/mL were incubated with RBCs in PBS (pH 7.4) after centrifugation. The solution did not appear any detectable change in color, while the RBCs incubated with DW were lysed and the supernatant turned red color. In contrast, there was no detectable red color in the supernatant of the RBCs incubated with the PPE-AgNPs that were further measured by spectrophotometer.

**Figure 9:**
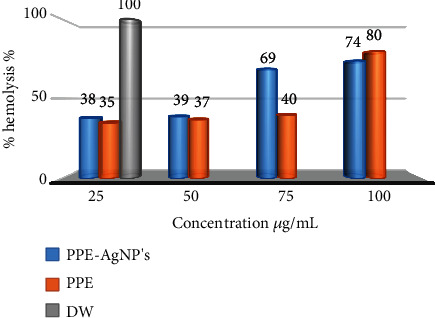
The hemolytic activity of PPE-AgNPs using concentrations 25, 50, 75, and 100 *μ*g/mL as compared with PPE and DW.

RBC hemolysis was demonstrated by the RBC integrity membrane breakdown that leads to hemoglobin release into the peripheral blood plasma [45]. For PPE, the in vitro hemolytic action for the concentrations 25 *μ*g/mL was 35%, while for 50 *μ*g/mL was 37%, and for 75 *μ*g/mL was 40%. For PPE-AgNPs, the concentrations 25 and 50 *μ*g/mL have indicated 38% and 39% of hemolysis, respectively, whereas 75 and 100 *μ*g/mL concentrations showed a significant effect on RBC hemolysis with 69% and 74% of hemolysis action that could reflect the nonhemolytic agent of PPE-AgNPs, as displayed in Figure 9.

**Figure 10:**
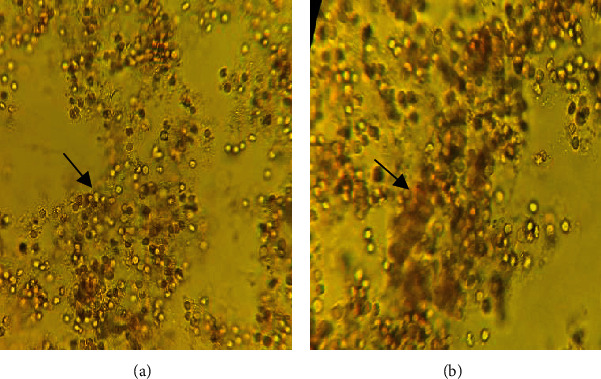
Microscopic investigation for erythrocyte morphological change when treated with (a) PPE-AgNPs and (b) distilled water.

Afterward, the biofabricated nanomaterials are ecofriendly methods. The particle components are biocompatible in nature and can be used as the anticoagulant agent for nanomedical applications. A previous report that demonstrated the antithrombic action of nanosilver concluded that these nanoparticles could inhibit the integrin-mediated platelet effect [46].  Figure 10(a) illustrates that RBCs treated with nanosilver did not show an effect on the membrane integrity that remained intact.  Figure 10(b) illustrates that the RBCs were treated with DW, it was lysed, and their hemoglobin may leak out as well as change in their morphology, which was confirmed by a light microscope, this result suggesting the PPE-AgNP's biocompatibility.

**Figure 11:**
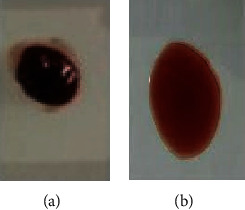
Visual results of anticoagulant activity of PPE-AgNPs. (a) The clotting of distilled water–treated human blood and (b) PPE-AgNPs–treated human blood (25 *μ*g/mL).

The anticoagulant properties of biosynthesized PPE-AgNPs were studied, and there was no blood clot observed in the PPE-AgNPs–treated blood sample. However, coagulation was immediately noticed in DW-treated blood (Figure 11). This confirms that the biosynthesized nanosilver can be used as a blood anticoagulant agent.

This is in accordance with the results obtained by [35]. A study proved the heparin anticoagulant effect when treating earthworms with nanogold particles [23]. In this report, it was proposed that nanosilver may possibly be able to inhibit blood clotting when preventing the fibrinogen for fibrin formation. Till now, it is not yet known which factor can inhibit the clotting of blood [47]. The biosynthesized nanosilver could be inhibiting platelet formation similar to the aspirin drug when it accumulates and inhibits blood clotting with different actions [48, 49]. The biogenic production of nanosilver with potential anticoagulant action increases attention to minimizing the usage of traditional anticoagulants. The plant extract–based nanobiofabrication was preferred as a natural, ecofriendly, and low-cost technique [50]. To enhance the efficacy-related concerns and safety, pharmacodynamics and pharmacokinetics for biosynthesized nanosilver need to be studied via clinical trials prior to their translation to therapeutics as anticoagulant agents.

## 4. Conclusion

The current in vitro study proved that pomegranate peel waste extract is an efficient reducing and capping agent for the biosynthesis of nanosilver with a size range of 20–30 nm and exhibited optical absorption properties by UV–vis analysis at 360–460 nm. The PPE-AgNPs were spherical in shape, and EDX analysis identified the silver element at 3 keV. The cubic crystal nature of nanosilver was confirmed by XRD. By AFM, the biofabricated AgNPs have a diameter of around 7.20–14.80 nm with a median of 7.16 ± 1.3 nm, and the RMS value corresponds to 1.40 ± 0.4 nm. Antioxidant properties of biosynthesized PPE-AgNPs efficiently exhibited a dose-dependent DPPH inhibition activity. PPE-AgNPs have indicated no demonstrable hemolytic activity which proved the biocompatibility as well as anticoagulant action. The combination of biowaste with green synthesis is more beneficial than other approaches for nanosilver fabrication thru ecofriendly, simple, one-step procedures, high stability techniques, as well as cost-effectiveness technique as well as PPE-AgNP's biocompatibility and anticoagulation action in the near future after *in vivo* validation.

## Data Availability

Data sharing is not applicable to this article as no new data were created or analyzed in this study.
